# Hydroxyapatite deposition disease of the wrist with intraosseous migration to the lunate bone

**DOI:** 10.1007/s00256-021-03758-z

**Published:** 2021-03-13

**Authors:** Sophia S. Goller, Nina Hesse, Hans Roland Dürr, Jens Ricke, Rainer Schmitt

**Affiliations:** 1 Department of Radiology, University Hospital, LMU Munich, Munich, Germany; 2 Musculoskeletal Oncology, Department of Orthopaedic Surgery, Physical Medicine and Rehabilitation, University Hospital, LMU Munich, Campus Grosshadern, Munich, Germany

**Keywords:** Bone marrow oedema, Carpal tunnel syndrome, Hydroxyapatite, HADD, Lunate bone, Wrist

## Abstract

Hydroxyapatite deposition disease (HADD) is a mostly uniarticular, self-limiting condition caused by deposition of hydroxyapatite (HA) crystals in tendons or in the peritendinous soft tissues. Commonly, the glenohumeral joint is affected. More rarely, the HA depot can be cause of a carpal tunnel syndrome due to an acute inflammatory reaction and space-occupying soft tissue oedema. We report a case of acute HA depot located at the volar site of the right wrist with affection of the deep flexor tendons and intraosseous migration into the lunate bone in a 50-year-old female. There are two main goals of this case report: First, to remind the diagnosis of HADD as a cause of wrist pain and also of carpal tunnel syndrome, as this entity being often misdiagnosed clinically, and second, to report a rare case of intraosseous migration of HA crystals into the lunate bone.

## Introduction

HADD is a cell-mediated inflammatory condition and characterized by periarticular depots of HA crystals within or around a tendon [1]. The pathogenesis of HADD is not yet fully understood; however, repetitive microtraumata and local ischemia are mainly thought to be responsible for the development of HA depots. Furthermore, association with other diseases such as end-stage renal failure, collagenosis or familiar tumorous calcinosis is being discussed [2]. The average age of onset is usually around 45 and women are more commonly affected than men (2:1) [3].

By far, the most common manifestation of acute HADD is the glenohumeral joint, with HA depots in or around the supraspinatus tendon in about two-thirds of cases [4, 5]. In this regard, osseous involvement in the context of HADD of the glenohumeral joint was summarized in a previous publication by Flemming et al. [6]. Less frequent manifestations are the hip joint, elbow, wrist and knee joint [7–9]. Affecting the wrist, HA depots are found preferentially in the tendon of flexor carpi ulnaris muscle proximal to the pisiform bone and at the metacarpophalangeal and proximal interphalangeal joints [10]. The natural course of HADD follows four stages: precalcific, calcific, resorptive and postcalcific [3]. In most cases, the HA inflammatory process is self-limiting and resolves spontaneously, but sometimes continued pain and functional impairment can occur either due to a lack of resorption of HA depots causing impingement-like symptoms or when liquified HA leaks from the tendon into the peritendinous soft tissues, causing a painfully local acute inflammatory reaction [2, 11, 12].

While there are several previous case reports presenting HADD as a cause of carpal tunnel syndrome [7, 13, 14], in our case, a rare observation of intraosseous migration of HA crystals into the lunate bone was made.

## Case report

**Fig. 1:**
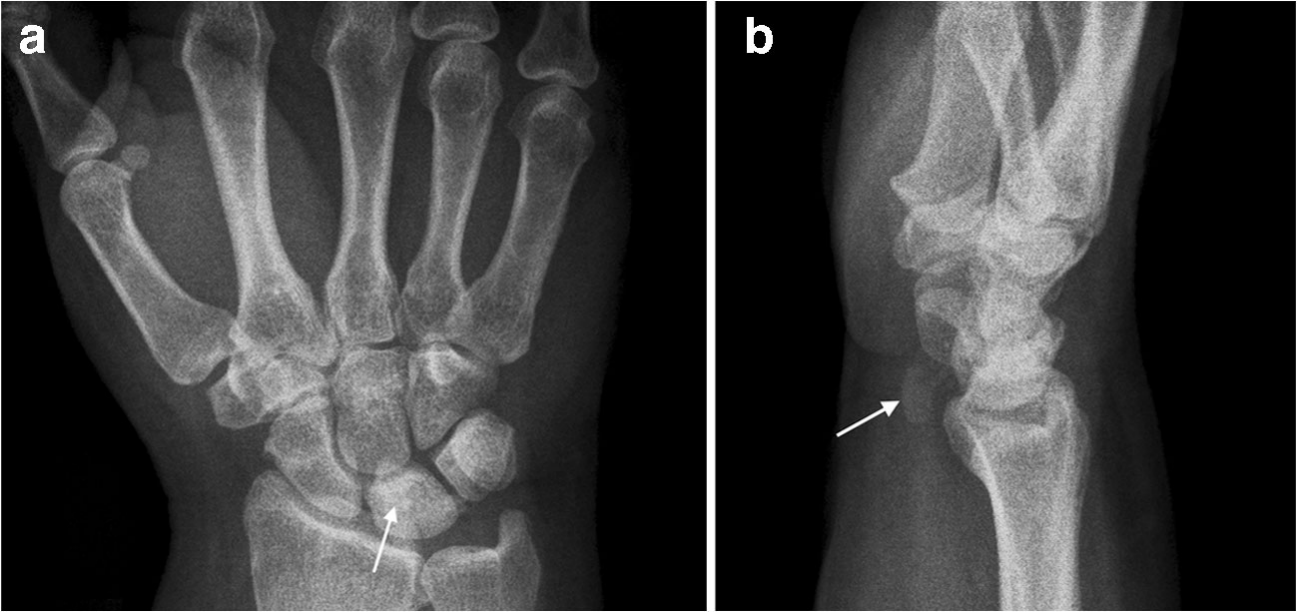
Radiographs demonstrating a solitary amorphous calcification at the volar site of the right wrist adjacent to the proximal carpal row (**b**, arrow). There are no signs of erosions or carpal malalignment. Note the slightly sclerotic lunate bone (**a**, arrow)

A 50-year-old female presented with a 4-month history of right wrist pain. The patient denied any history of fever, trauma or previous surgery. Physical examination showed slight swelling and warmth, while skin appearance was normal. Range of passive and active motion was painful, but not restricted. However, palpation of the wrist revealed a local pressure pain above the lunate bone. In addition, she presented mild symptoms of carpal tunnel syndrome like slight tingling and numbness, but no weakness. Bottle sign was positive. Plain radiographs revealed a solitary, oval-shaped, amorphous calcification within the volar soft tissues adjacent to the proximal carpal row (Fig. 1). The lunate bone was slightly sclerotic compared to the other carpal bones, which were all normal in shape. There was no carpal malalignment and no findings concordant with erosions. In accordance with clinical and radiological findings, the working diagnosis of HADD was made.

**Fig. 2:**
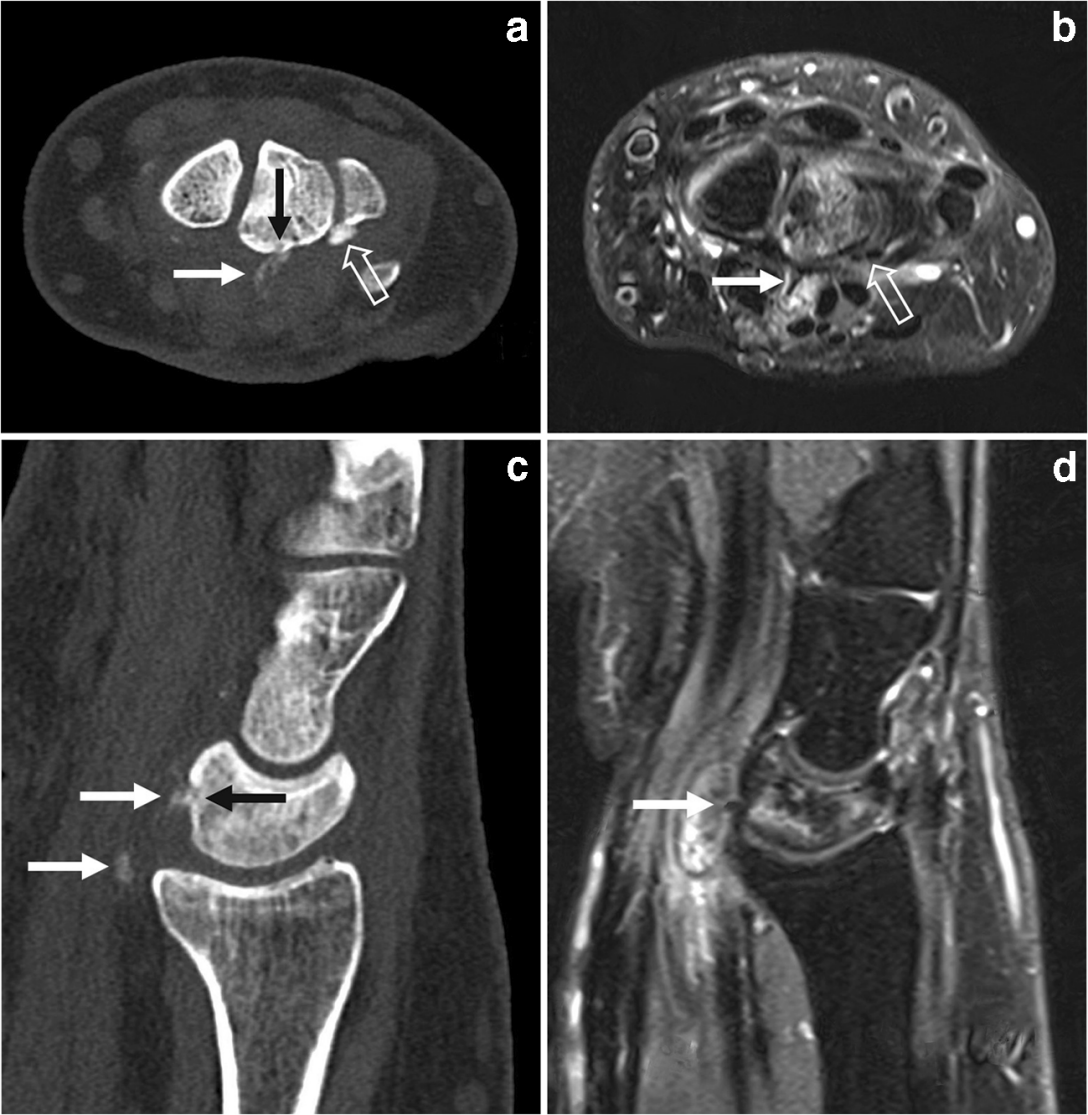
Transaxial and sagittal CT and MR images. **a** CT image with two calcified structures of different density. The tail-like, amorphous calcified depot (white arrow), consistent with already liquified HA shows intraosseous migration into the lunate bone. A small erosion is evident (black arrow). A dense, smooth-edged calcification depot located at the lunotriquetral junction is concordant with a calcific HA stage (open arrow). **b** In fat-saturated proton density–weighted MRI, both calcifications (white and open arrows) appear hypointense. They are surrounded by soft tissue oedema. An inhomogeneous bone marrow oedema is evident in the lunate bone. **c** In the sagittal CT image, loosened calcifications are present in the depth of the carpal tunnel (white arrows). There is an erosion at the cortex at the anterior horn of the lunate bone (black arrow). The small erosive defect is filled with calcified depots. **d** In sagittal MRI (fat-saturated proton density–weighted), the calcifications are just visible as hypointense spots, one of them located directly at the anterior horn of the lunate bone (white arrow). In the carpal tunnel, there is a space-occupying oedema of soft tissues with volar displacement of the deep flexor tendons. The bone marrow oedema of the lunate bone is patchy and inhomogeneous

For further evaluation, CT was performed to better locate the HA calcifications within the carpal tunnel and to assess bony changes of the lunate (Fig. 2a and c). CT scan showed two calcified structures of different density: First, an oval-shaped, amorphous calcification at the volar site of the wrist within the soft tissues, already known from the radiographs. This calcified structure was consistent with a liquifying HA depot in the resorptive stage and acute soft tissue inflammation. Additionally, an intraosseous migration of the HA crystals into the lunate bone was evident (Fig. 2c).

Second, there was a small, smooth-edged, very dense calcification depot located at the ulnar site of the lunate bone, which was consistent with a HA depot in the calcific stage (Fig. 2a). The lunate bone was normal in shape but appeared slightly sclerotic compared to the adjacent carpal bones, consistent with the radiographs.

Additionally, MRI was performed (Fig. 2b and d) visualizing an extensive inflammatory process around the liquifying HA depot within the deep flexor tendons and the adjacent soft tissues. There was a nearly territorial bone marrow oedema of the lunate bone indicating an intraosseous inflammatory process. However, no inflammatory process was seen around the dense HA depot in calcific stage.

Based on clinical and radiological findings, the diagnosis of HADD-associated carpal tunnel syndrome was made and treatment with non-steroidal, anti-inflammatory drugs (NSAID) was started. The patient’s symptoms decreased within 2 weeks. Surgical release of the retinaculum flexorum was therefore not necessary.

## Discussion

To our best knowledge, this is the first report describing a case of HADD-associated carpal tunnel syndrome with intraosseous invasion of HA depots at the wrist. Carpal tunnel syndrome is either caused by a volume increase of its content or by a decrease of the tunnel volume itself [15]. In rare cases, crystal deposition diseases like calcium pyrophosphate deposition disease (CPPD), gout and HADD may become space-occupying in the carpal tunnel, particularly when liquified crystals leak from the tendon into peritendinous soft tissues causing flexor tenosynovitis [15, 16]. In the acute inflammatory phase, the median nerve is compressed with sensory symptoms being triggered [7, 13, 14].

In the majority of cases, the diagnosis of HADD is made by physical examination as well as by radiographs, which demonstrate the more or less dense calcifications at the site of involvement [9]. HADD usually follows four stages: In the precalcific stage, the HA crystals begin to get organized around the affected structure. In the calcific stage, HA crystals can be detected as dense, uniform calcifications on radiographs. In the resorptive phase, calcifications start to liquify and break out of the tendon leading to an acute clinically painful phase due to inflammatory tenosynovitis. Then, calcifications are less dense on radiographs and CT. At last, in the postcalcific stage, the calcifications disappear [17, 18].

As HADD in the acute inflammatory phase goes along with soft tissue inflammation and oedema, it might be misdiagnosed, e.g. as cellulitis, infectious arthritis or gout [17]. In addition, there are some important differential diagnoses in particular the bone marrow oedema of the lunate, which can usually be resolved radiologically based on the distribution pattern of the oedema, lunate and carpal morphology, ulnar variance, as well as clinical symptoms [19, 20]: (a) Kienböck’s disease is often associated with ulnar minus variance, proximal necrosis zone and radial localized bone marrow oedema. (b) Ulnar impaction syndrome shows signal alterations at the ulnar side of the lunate, usually positive ulnar variance, and triangular fibrocartilage complex (TFCC) perforation. (c) Intraosseous ganglia are localized at the insertions of the scapholunate or lunotriquetral ligaments as sharply circumscribed and marginally sclerosed cysts either on the radial or ulnar side of the lunate. (d) Lunotriquetral synchondrosis shows an irregular joint space and signal alterations at the lunate and triquetrum. (e) Cortical avulsion fractures and fractures through the body of the lunate are clearly identified by CT. (f) Perilunate osteoarthritis typically omits the radiolunate compartment and manifests distally at the lunate as mediocarpal osteoarthritis. (g) Rheumatoid arthritis and other spondyloarthropathies usually cause erosions also in other localizations of the hand. Important for differentiation, all of the abovementioned entities are not accompanied by calcifications in the soft tissues. In this regard, the main differential diagnosis remains CPPD arthropathy, which is encountered much more frequently at the wrist than HADD. Features of CPPD arthropathy are linear, usually multilocular calcifications in articular cartilage, TFCC and synovia. Carpal collapse, signs of osteoarthritis and multicystic lesions occur in advanced stages of carpal CPPD wrist arthropathy.

In our case, we did not perform a biopsy procedure with crystal analysis for the following reasons. First, the patient did neither have elevated leukocytes or uric acid levels, nor did she have a history of gout. Second, all imaging features were HADD-typical and other crystal-associated arthropathies could be excluded by means of imaging due to different morphology of calcifications. Unlike with CPPD, a HA depot usually presents as solitary calcification spot with an amorphous structure and is found in the course of tendons, but not in cartilage tissues. HA depots are thus in contrast to the linear, granular or crumbly CPPD depots [19]. Third, since the patient responded well to the antiphlogistic therapy with NSAIDs, surgical treatment was not necessary.

This report describes a rare case of HADD of the deep flexor tendons in the carpal tunnel and intraosseous HA migration into the lunate bone with intense bone marrow oedema. From our point of view, the HA crystals have migrated into the lunate via a small cortical erosion located at the anterior horn of the lunate 3 mm radial to the nutritial canal at the level of the radiolunate ligaments. Most likely, the long radiolunate ligament is the anatomical structure along which the HA crystals migrated, as this ligament originates at the palmar side of the scaphoid fossa at the distal radius, runs to the anterior horn of the lunate, where it has an attachment, and then continues to the triquetrum. The short radiolunate ligament is much less likely to be assumed in our case, as its attachment site at the anterior horn of the lunate is located more proximal [21].

In a previous study, inflammatory response with focal hypervascularity at the site of tendinous involvement was discussed being responsible for local bone resorption at the osseous junctions with consecutive intraosseous migration of HA crystals in different manifestations [6]. According to current knowledge and our own experience, osteotendinous junctions seem to be the predilection sites for intraosseous migration of crystals in HADD [1, 6, 22]. In our patient, the HA crystals most likely did not migrate via the nutritial canal into the lunate; nevertheless, a possible explanation for the sclerotic aspect of the lunate bone might be an impairment of blood supply by intraosseous HA crystals, which might lay the entry point of the nutritial vessels from the intraosseous side.

Oral NSAIDs are usually the first-line treatment to alleviate pain and shorten the acute inflammatory phase. Other therapeutic options include oral corticosteroids or ultrasound-guided calcific lavage, in cases of calcifications over 1 cm [17]. The image-guided injection of local corticosteroids into the carpal tunnel directly next to the median nerve may be taken into account to dissolve the calcific depots. The injection procedure is ultrasound-guided via a radial or ulnar approach, with the ulnar approach being currently preferred [23–25]. In non-responders surgical release of the carpal tunnel should be considered.

## Declarations

Written informed consent was obtained from the patient included in this study.
